# Taxing the rich: public preferences and public understanding

**DOI:** 10.1080/13501763.2021.1992485

**Published:** 2021-12-13

**Authors:** Lucy Barnes

**Affiliations:** Department of Political Science, University College London, London, UK

**Keywords:** Ideas, public opinion, taxation

## Abstract

Who supports high taxes on the rich? Existing accounts of public attitudes focus on egalitarian values and material interests, but make little mention of the ideas people hold about how the economy works descriptively. Drawing on the distinction between positive- and zero-sum beliefs about the economy, and original survey data from five countries, I show that there are systematic differences in tax progressivity preferences across groups within the public who think differently about the economy. Positive-sum thinking is associated with less progressive preferences. However, despite theoretical attention, there is no evidence of systematic zero-sum thinking among the public. On the other hand, some descriptions focus on conflict between rich and poor, and these do predict support for greater progressivity. Further analysis is required to differentiate alternative causal explanations of the patterns observed, but different modes of descriptive economic thinking are an important feature of the mass politics of progressivity.

## Introduction

Who supports taxes on the rich? This question is central to the politics of taxing the rich. Public opinion shapes the political consequences of any proposal. As such there exists a large literature explaining individual preferences over redistribution and taxation. In these accounts, material interests loom large: richer segments of the population are typically less supportive of progressive structures, which would see them liable for higher payments (Beramendi & Rehm, 2016). But the emphasis given to equality as a value is also taken seriously, for example when war changes norms around fairness (Scheve & Stasavage, 2015; Walter & Emmenegger, 2021). Many scholars argue more broadly that it is in normative values that some of the most interesting dynamics are to be found (Cavaillé, 2021).

However, these accounts pay little attention to descriptive ideas: ‘beliefs about cause-and-effect relationships’ that provide actors with expectations about policy consequences (Lindvall, 2009, p. 704). These descriptive beliefs are an important complement to interests and normative ideas. No matter how strong my egalitarianism, nor how low my income, I will not support highly progressive taxation if I believe it will stifle all economic activity, or lead to massive evasion by the rich. This is a caricature, but we know that descriptive ideas among elite actors influence tax policy decisions (Steinmo, 2003). Why not public attitudes?

I examine the presence of three broad profiles of economic ideas in nationally representative surveys from Denmark, France, Germany, the United Kingdom and the United States. Following recent literature in economic psychology, I consider the prevalence of a zero-sum mentality, wherein economics is a distributive battle over one’s share of the pie, contrasted with positive-sum thinking, which focuses on the size of the pie (Rubin, 2018). Finally, recent work in political science, motivated by a perceived disconnect in political communication on economic issues, highlights a third mode of economic thought. In these accounts, popular thinking on the economy is characterised by a lack of engagement with formal descriptions of macroeconomic phenomena (Killick, 2020).

These descriptive ideas should be consequential for attitudes towards taxing the rich. The zero-sum mentality is highlighted by Rubin (2003) as focused on ‘the distribution of taxes, not the implications as incentives for behavior [sic]’ (p. 165). In contrast, the positive-sum focus on overall efficiency is the same set of ideas seen to underpin late-twentieth-century moves away from progressive taxation in policy (Steinmo, 2003). Finally, disengagement from thinking about the economy at the macro-systemic level tends to be found among the same people who perceive the economy to be rigged by the rich, including through their evasion of taxes (Killick, 2021, p. 11).

I thus ask three central questions in this paper. First, how prevalent are these different types of beliefs in popular economic thinking? This is important, because even if a zero-sum mentality were closely linked to support for progressive taxes, its relevance to the politics of taxation scales in proportion to its presence in the population. Second, who espouses zero-sum, positive-sum, or disengaged thinking? This matters for the politics of taxation to the degree that the different groups have differing degrees of political voice. Finally, are these broad descriptive models of how the economy works systematically linked to differences in preferences over progressivity? The role of these cause-and-effect beliefs may be an under-examined component of public opinion on taxation.

To examine these ideas, I use latent class analysis to identify specific types of people in terms of their zero- or positive-sum views, or non-engagement responses. Interestingly, zero-sum thinking does not seem to characterise the thinking of any appreciable share of the population. Second, even taking material interests and explicitly articulated beliefs about the value of equality into account, the different descriptive frameworks are systematically associated with preferences over progressivity. In particular, a small group with strongly positive-sum views of economics are particularly averse to progressive taxes.

## Types of economic thinking

How do the public think about the economy, and economics, in ways likely to be relevant for views about taxing the rich? The most general accounts of popular economic thinking come from two sources: economic psychology on the one hand, and interpretive political economy, on the other. The former tends to focus on ‘folk economics’: how it diverges from expert, scientific economics, and what explains these folk beliefs. The latter tends not to privilege expert economics in normative terms, but to describe the views of the public from the bottom up. However, a central motivation in this literature is the perceived disengagement of citizens from economic debates, news, or concepts (Killick, 2021). I take these general frameworks as my point of departure.

One possible way of thinking about the economy is that of the classical economist. In this thinking, prices communicate incentives and behaviour responds, shaping the overall size of economic activity. These indirect consequences mean that ‘[e]conomists reject [a] fixed-pie mentality’ (Caplan, 2007, p. 65) in favour of the idea that economic transactions are positive-sum.

However, economists and the public do not think alike (Boyer & Petersen, 2018; Sapienza & Zingales, 2013). In particular, Rubin (2003) argues that zero-sum thinking as a general framework underpins many specific features of popular (mis)understanding:

folk economics is the economics of wealth allocation, not production … each individual is concerned with the distribution, … not with any efficiency gains from economic activity. The world … is a zero-sum world. (Rubin, 2003, p. 157)

This zero-sum view is defined by its neglect of indirect behavioural responses that may increase the size of the pie.

Finally, many people may not consider themselves to think about the economy descriptively at all. Killick (2020) finds that many people understand the economy only very locally, with few points of connection to an abstract ‘economy’. In addition to the distinction between positive- and zero-sum thinking, there may be a profile which is primarily characterised by non-engagement.

### Public economic ideas and tax policy preferences

On the positive-sum idea that markets provide the framework for mutually beneficial exchanges, interventions which reduce these exchanges will mean missing out on these gains. High marginal tax rates on the rich may lead to large distortions of this kind. Thus, the positive-sum view should reduce support for progressivity. In contrast, without attention to dynamic effects via incentives, zero-sum thinkers will not recognise adverse behavioural effects of the intervention, and thus express a greater level of support for progressive taxation.

Economic non-engagement might extend to lacking specific views on taxation. However, three lines of thinking lead to the expectation that people who are disengaged from thinking about the economy may be more supportive of progressivity (than the positive-sum thinkers, at least). To the extent that positive-sum economic thinking leads to greater scepticism of progressive taxation, or is learned from elites who also push for tax cuts on the rich, those who are disengaged from economic thinking would be more supportive of progressivity due to lack of exposure to these ideas. Second, non-engagement with economic thinking may be a response to an economy seen as rigged in favour of the interest of the rich (Killick, 2020). To address this imbalance, the non-engaged might advocate progressive taxation. Finally, non-engagement in economic thinking may result from a divergence between people’s preferences and mainstream discussions. If positive-sum economic ideas dominate elite discourse, those with more progressive tax orientations might disengage from economic thinking *because of* these preferences.

Describing public thinking on the economy with reference to the overarching frameworks of positive and zero-sum thinking may generate scepticism based on the large literature in political psychology stressing that only a minority of the population hold stable, coherent ideological positions (Zaller, 1992). However, the approach here is to identify shared patterns of response across respondents empirically, rather than specifying coherence across variables on the basis of their content. Once groups are identified by their collective similarities, the ‘Zallerian elite’ may be more distinctive in the content of their economic thinking, or in the strength of its links to tax preferences.

### Zero- and positive-sum thinking and other descriptive beliefs

Studying descriptive ideas in popular thinking extends the analysis of economic ideas beyond the elite level, where political economy scholarship has been highly attentive to the role of ideas. Much of this work focuses on the rise of neoliberal ideas (Blyth, 2002), and the transmission of ideas from the discipline of economics (Fourcade, 2009). Importantly, the salience of neoclassical concerns with the efficient production on the supply side has increased (Mudge, 2018). Greater attention to incentive and growth effects, and a downgrading of the relative salience of distributive questions represents a rise of the positive-sum mindset in the terminology of this paper.

Zero- and positive-sum beliefs, even with the complementary position of non-engagement, do not encompass the full space of possible descriptive beliefs. But these three ways of thinking are general, systemic, organising descriptive principles. It is possible to cast many more specific descriptive beliefs as instances of zero- or positive-sum tendencies. For example, beliefs about the incentive effects of taxation can be readily incorporated into the zero- versus positive-sum distinction, as responsiveness to incentives is the mechanism that generates positive-sum possibilities.

However, not all specific factual descriptive beliefs about the economy are well-accommodated within this theoretical framework. One such type of belief concerns perceptions of inequality and economic mobility (McCall, 2013). Beliefs that hard work leads to economic success, for example, is compatible with a zero-sum view (this success is at the expense of others, albeit others who work less hard). But it is equally compatible with the idea that the additional hard work or talent grows the pie and benefits everyone. As such, while mobility beliefs are another set of descriptive beliefs which may affect preferences over tax progressivity, they fall outside the scope of this paper.

Similarly, the three models outlined above do not in themselves incorporate judgments about justice or deservingness. For example, I might see the economy as a zone of conflict between rich and poor but nevertheless think that the rich deserve all that they achieve within that conflict. Some of the links between beliefs about the causes of economic success and preferences over inequality and redistribution highlight the significance of these descriptive beliefs because of the normative conclusions that they tend to imply (Suhay et al., 2021). This kind of description – of the morally relevant characteristics of those who might gain or lose from progressive taxation – is another distinct type of belief which is already well-documented, in particular in work on fairness (Cavaillé, 2021).

## Descriptive empirics

The data for these analyses come from an original survey fielded in Denmark, France, Germany, the United Kingdom and the United States in June and July 2020. The sample comprises approximately 1000 respondents per country from Respondi’s online access panel.^1^ In each country, the sample is representative of the national population in terms of age, gender, and regional distribution.^2^

I use a battery of six questions based on Rubin (2003)'s characterisation of zero-sum economic thinking, contrasted to the positive-sum view, that we saw above. Specifically, I ask about whether economics and economic policy are concerned with the distribution of existing resources, or growing the overall size of the pie.^3^ Rubin also specifically characterises the lump-of-labour fallacy as indicative of zero-sum thinking, so a second pair of items proposes zero- versus positive-sum accounts of the labour market. Finally, I use two items focused on whether increases in the incomes of the rich are beneficial or harmful overall. These questions highlight Rubin's distinction between a fixed amount of total output and a growing one, via two different framings of the successes of the rich. The items on the rich are taken directly from the ‘belief in a zero-sum game’ battery (Rózycka-Tran et al., 2015) used in broader (beyond economic) psychological applications.

I estimate latent class models to categorise respondents into classes of economic thinking on the basis of this battery. Latent class analysis is a method for the analysis of categorical outcome data which assumes an underlying categorical structure to the latent variables shaping profiles of response. Algorithmically, the analysis proceeds by stipulating the number of latent classes, and then optimally separating the data on the assumption that, conditional on class membership, response outcomes are independent. It seeks to allocate variation in the measured variables entirely between classes rather than within them. The latent class model will ‘identify and characterise clusters of similar cases, and approximate the distribution of observations across the many variables of interest’ (Linzer, 2011).

Why latent class analysis? The primary reason is substantive, and based in the fit between theory and methodological approach. Specifically, the substantive question is whether we can identify citizens who embody particular patterns in terms of their economic thinking. The central descriptive question is at the respondent level, rather than the variable level. Thus while the variance-minimization problem is analogous, it is theoretically more appropriate to look for latent classes of people than to describe variables which characterise dimensions of economic thinking. This approach to identifying types of citizens is common in political science (e.g., Bertsou & Caramani, 2020).

Second, the logical structure of the types of thinking anticipated points to the classification of latent types, rather than continuous latent spaces. Specifically, non-engagement with economic thinking logically separates people from any kind of thinking about these questions. It is not a particularly sensible question to ask how zero-sum the thinking of someone who is disengaged is. Only those who engage can be described in terms of this attribute.

Finally, using the latent class approach allows me to leverage non-response in the battery to describe those who disengage from economic thinking. Ex ante, the advantage is to be able to study ‘don't know’ answers as substantive responses amenable to subsequent analysis. A ‘don't know’ group cannot readily be included in analyses based on an index, or on factor analysis, whereas the categorisation approach focusing on response profiles allows us to identify people who give multiple don't know responses as a natural and interesting group. As it turns out, this also provides a useful mechanism for identifying profiles of other kinds of non-informative response patterns: acquiescence and non-commitment (always selecting ‘neither agree nor disagree’). This was not anticipated before the fact, but means that the latent class approach allows for a much richer description of the full population.

To allow for the unbiased estimation of the relationships between respondent characteristics and class membership, I estimate latent class regression models including covariates (country, age, gender, education and income). Separate estimation of the links between covariates and classes tends to underestimate the size of these relationships (Bolck et al., 2004). In this application, including the covariates, especially income quintile, also improves model fit. However, models without covariates yield similar substantive results.^4^

The latent class model requires the number of classes to be specified. Selecting the right number of classes depends on a combination of prior theory, empirical fit as measured by model information criteria, and the interpretability of the resulting classifications.^5^ The AIC continues to fall through eight classes, while the BIC (more conservative with regard to overfitting) is minimised at seven. The eight-class solution is characterised by five very small and unstable (across starting values of the priors) classes, and much less readily interpretable patterns of responses within classes than the seven-class solution. Analysis with six classes yields a similar solution (to seven), but combines the two largest groups into a single category (incorporating almost half the respondents) with little other change. I proceed with the seven-class model.

### A description of public economic thinking

The latent class regression divides the survey sample into seven classes which do not map neatly onto a simple ‘more or less zero-sum thinking’ dimension. This emphasises the value of considering response profiles as a whole. There are three distinct types of non-engagement, two groups who can be broadly be said to be positive-sum thinkers, but no consistent zero-sum thinking group. Instead, we see two groups who are distinctive in their attention to questions of distribution.

**Figure 1.**
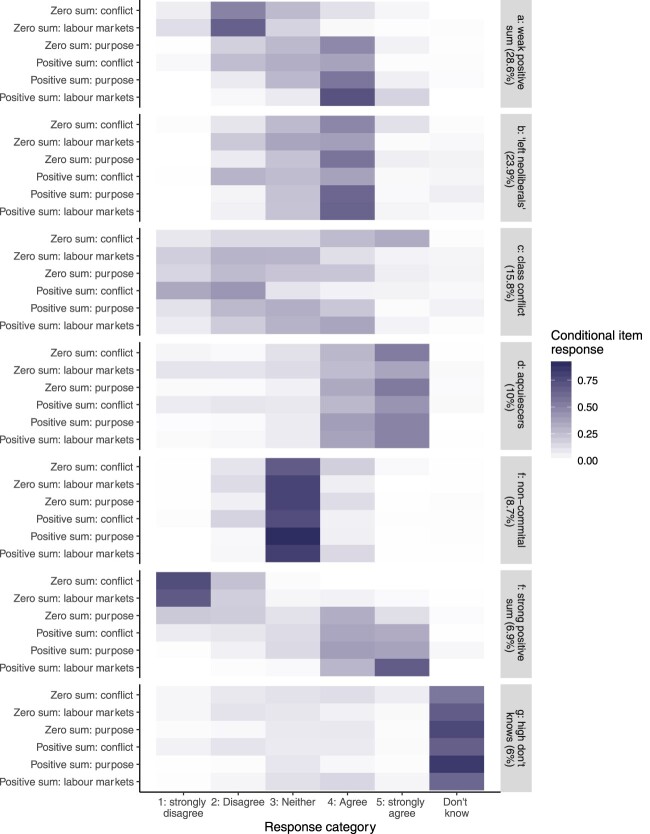
Latent class profiles: seven class model with covariates: country, age, gender, education and income.

Figure 1 summarises the analysis. Each panel in the figure illustrates the response shares for each category for each question, conditional on membership within a class. Classes are ordered by their estimated shares in the population. Given the ordering of the questions (zero-sum valence questions at the top of each panel, and positive-sum valence questions at the bottom) and the responses (ordered left to right from disagreement to agreement) a zero-sum profile would be characterised by high shares (dark colours) on the southwest–northeast diagonal in each panel. Positive-sum thinking concentrates responses on the northwest–southeast diagonal.

The most common profile in the data shows weakly positive-sum economic thinking (top panel of Figure 1). This group, 29 per cent of the population, combines disagreement with the zero-sum views of distributive conflict and labour market competition with agreement that economics is about questions of growth, and that employment is a positive-sum phenomenon. I characterise this group as ‘weakly’ positive sum because these agreements (and disagreements) are not ‘strongly’ endorsed, and because they broadly endorse the importance of distribution as part of the purpose of the economy, and are ambivalent about positive spill-overs from the incomes of the rich.

The second-largest category in the survey population comprises a quarter of respondents, with generally moderate (little ‘strong’ (dis-)agreement) responses. They agree with the ‘zero sum conflict’ item (where the weak positive-sum thinkers disagreed), but simultaneously endorse positive-sum views of the labour market and tend to reject lump-of-labour thinking. This balance between distribution and conventional economic efficiency commitments is also reflected in this group's simultaneous endorsement of both the distributive and growth-oriented framings of the purpose of economics. As a reflection of this dual commitment, I label this group ‘left neoliberals’.

The response profiles of the two remaining ‘engaged’ categories are shown in the third and sixth panels of Figure 1. The first, I label the ‘class conflict’ group: they strongly agree that distributive conflict is zero-sum. However, beyond the conflict items, the picture is not one of straightforward zero-sum thinking. The positive-sum view of employment is weakly endorsed, and the zero-sum picture weakly rejected. Economic thinking in this group is better described as focused on distributive conflict than as generically zero-sum. Finally, 6.9 per cent of the population form a group with a strongly positive-sum profile: strong disagreement with the zero-sum views of distributive conflict and labour markets; strong endorsement of positive-sum dynamics in employment, and agreement that income gains at the top are positive sum, and growth is the purpose of economics. This class is the only one with any appreciable share of disagreement with the idea that economics is about the distribution of existing resources.

Finally, the latent class model differentiates multiple kinds of non-informative response in the data. There is the expected set of people who respond that they don't know for a large share of the items. However, there are two other response patterns which may indicate a lack of engagement with economic thinking: a group who tend to agree (and strongly agree) with all the items, and a group who neither agree nor disagree throughout. These classifications measure underlying economic engagement and engagement with the survey jointly, which limits the weight we should put on interpreting these responses as direct expressions of *economic* ignorance, acquiescence or indifference, but the groups are populated by different types of individual in the sample, and correspond in different ways to tax policy preferences.

The central descriptive finding here, then, is that there is little evidence of a consistent zero-sum mindset in popular economic thinking, despite its prominence in the economic psychology literature. While some people do endorse individual elements of a zero-sum mentality, this does not imply an organising zero-sum structure. In contrast, many respondents combine positive-sum logic (especially when thinking about the implications of employment, and the need to pursue a bigger pie) with a simultaneous commitment to questions of distribution, and the view that gains for the rich may come at the expense of others. On the other hand, a small group with strongly positive-sum views – with no perceived distributive conflict, nor need for attention to distribution – does exist.

#### Characterising the different classes

Who makes up these different classes? Table 1 describes the seven classes as classified by modal posterior probability, and the full sample, in terms of the covariates included in the model, and some other variables of interest, such as political attention.

**Table 1.** Class average characteristics based on posterior predicted classes from seven-class covariate model. Rows in grey indicate 95 per cent confidence intervals.

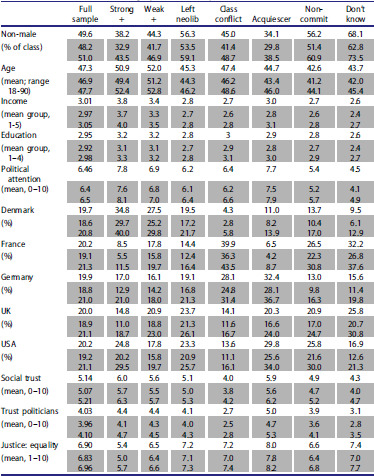

The data reveal some unsurprising patterns: the strong positive-sum class is richer, more male, older, drawn from higher education categories, and more politically attentive than average, as is the weak positive-sum group (to a lesser degree).^6^ Both these groups tend to have higher levels of social trust, and trust politicians to a greater extent. They put less emphasis on equality as necessary for justice. Meanwhile the left-neoliberal group is more female, younger, poorer, and drawn from lower education categories than the sample as a whole. They put more emphasis on equality, but are only marginally less politically attentive, and no different in terms of their levels of trust. The class conflict group is marginally male-dominated, poorer, and much less trusting than average. The non-committal and don't know categories are disproportionately female, younger, and poorer. Meanwhile, the patterns within the acquiescing group indicate that this group is most clearly identified by positive survey responses , as they also report high values on all the attitudinal measures (trust, equality, political attention).

The cross-country breakdowns are somewhat more surprising, with the positive sum groups drawing a disproportionate share of Danish respondents, and the class conflict group more French and German. British and American respondents are overrepresented among the left neoliberal group, which perhaps makes sense in the context of strong traditions of liberal economics in those countries, but also recent histories of increasing, and increasingly salient, distributive issues.^7^

## Descriptive ideas and tax policy preferences

These descriptive results point to taking slightly different expectations to the tax progressivity data than the theoretical discussion initially suggested. Specifically, since there is no zero-sum mindset in the data, it makes no sense to ask whether zero-sum thinking underpins support for progressive taxation. However, we would expect their emphasis on the conflict between rich and poor to lead the class conflict group to be particularly predisposed to endorsing progressive taxation. Similarly, the importance of distributive concerns for the left neoliberals might translate into support for progressive taxes, but this group holds some quite positive-sum views as well.

What about the three non-response groups? The acquiescer group's propensity to agree or choose high values for other survey items leads to the expectation of endorsement of progressive taxation in the survey, more on the basis of measurement artefact than substantive economic thinking. For the non-committal group, responses across the descriptive data do not necessarily indicate the same survey-wide tendency to choose the middle category, but to the extent that we see low endorsement of progressivity among this group, it may be due to a preference for the middle of the road response rather than a precise aversion to taxing the rich, given that average support for progressivity is high. For the group that tends to give ‘don't know’ responses, we would anticipate higher levels of don't know response on the tax preference question on the basis of both survey-response pattern and more substantive reasons. But to the extent that this group captures those disconnected from formal economic reasoning, we also expect them to more strongly endorse progressivity.

### Analysis of policy preferences

To measure preferences over tax progressivity, I ask respondents whether they think that people with high incomes should pay a larger or smaller share of their income in taxes than those with low incomes.^8^ Two things are worth noting. First, the question is phrased without reference to existing tax levels, to try provide a more general measure. Second, progressivity is extremely popular. 68 per cent of respondents think that those with high incomes should pay either a ‘larger’ or a ‘much larger’ share of their income in taxes than those with low incomes. A quarter of respondents endorse a proportional structure. These high levels of support for progressivity are in line with other estimates (Barnes, 2015). In the regression analyses, I use numeric values of support for progressivity which range from 1 (high incomes should pay a much smaller share) to 5 (a much larger share). Ordered and binary versions of the outcome yield similar results.

#### Analysis and results

**Figure 2.**
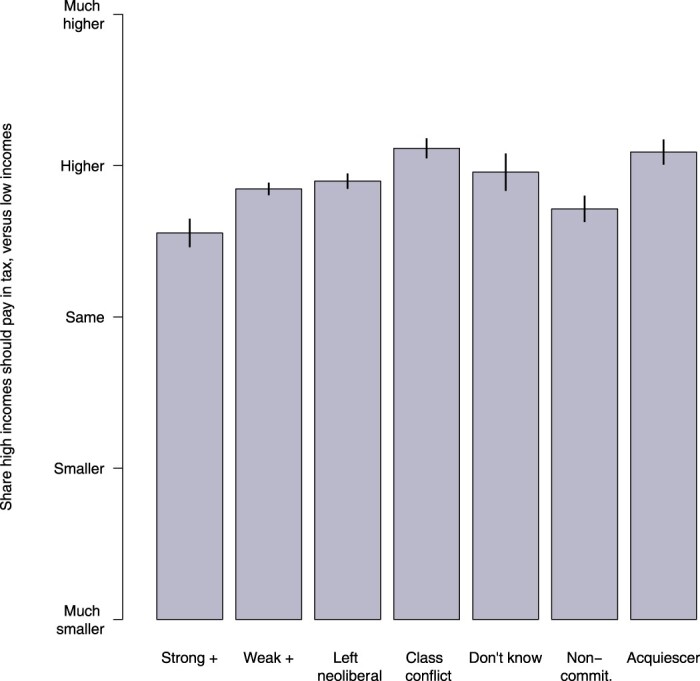
Average preferences towards progressive taxation across types of economic thinking. Mean tax outcomes by modal posterior class membership. Vertical black bars indicate 95 per cent confidence intervals.

I use a series of simple regression models to summarise the relationships between economic thinking and progressivity preferences, taking into account other associations. First, however, Figure 2 shows the average responses to the tax progressivity question for each group in the raw data.

We see significant differences across groups. The two positive-sum groups and the left neoliberal and class conflict groups appear in the order we would expect, with the class conflict group endorsing significantly higher progressivity, and the strongly positive-sum group much less. The don't know group average is higher than all these groups other than class conflict, while the non-committal group appears quite averse to progressivity. However, this may reflect concentration on the middle category among this class, just as the high progressivity rates among acquiescers is likely to be partly driven by the measurement tool. Identifying these specific patterns of non-informative response with the latent class analysis thus helps us to separate these high (acquiescer) or low (non-committal) reported levels which are more likely driven by measurement than substance, from the true expressions of those levels within other groups.

Regression analyses allow us to consider the robustness of these associations once other differences between the groups are taken into account. The different distributions of thinking across countries, and the interests and values highlighted in the existing literature, along with other individual characteristics, can be incorporated as control variables to investigate the associations between progressivity and economic thinking even conditional on these factors. I present models including an expanding set of covariates for country, demographics, material interest, values, and certain other political attitudes and behaviours.

**Figure 3.**
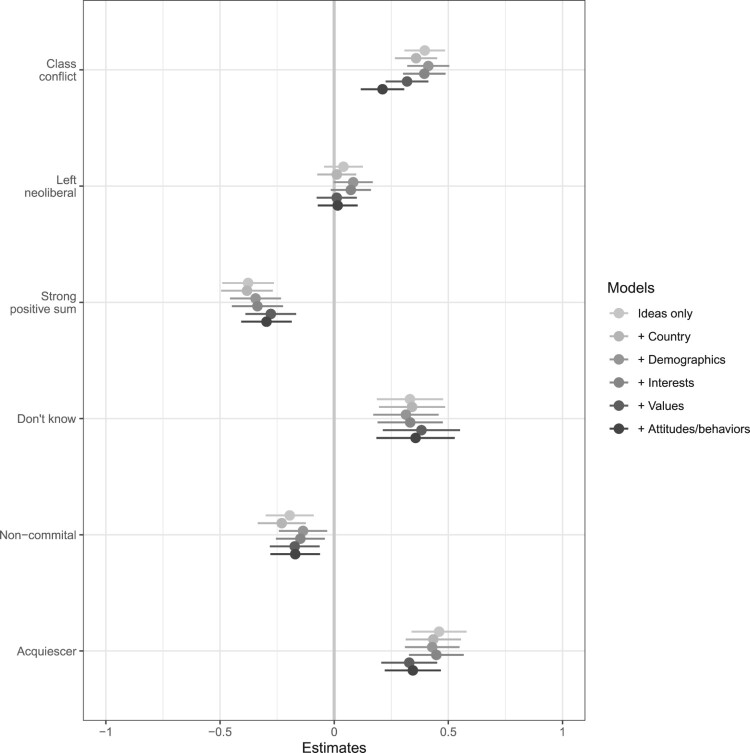
Estimates of the association between types of thinking and progressivity preferences relative to the weak positive-sum category. Estimates from six models: ‘ideas only’ includes only the posterior probabilities of membership in each of the latent classes; ‘Country effects’ adds indicators for country. Demographic controls are gender, age, labour force status, type of employment. ‘Interests’ adds covariates for income and education; ‘Values’ adds endorsement of equality as a requirement for fairness. ‘Attitudes/behaviours’ further adds social trust, trust in politicians, and political attention.

Figure 3 summarises these results.^9^ The estimates plotted are the regression coefficients from a set of models which take the weak positive-sum group as its baseline. Each point thus shows the estimated conditional difference between the named class and this baseline group. The models gradually add covariates, as labelled and discussed in the caption to the figure.

Note that the distinctive progressivity preferences of those who view the economy in class conflict, or strongly positive-sum terms, remains apparent across all specifications. In general, the differences across groups are relatively stable as we include covariates representing their interests and, to a slightly lesser extent, their values. The reduction in the class conflict estimate when controlling for attitudes and behaviours is due to the distinctively low trust in politicians that this group has, along with that variable's correlation with support for progressivity, and for the strong positive-sum group the (negative) relationship is diminished by including values in the model – recall that this group assigns distinctively little value to equality.

To understand the magnitude of these estimated effects, we can compare them to the differences between high- and low-income groups. The estimated difference of 0.2 points between the class-conflict group and the weakly positive-sum group (in the model with full controls), for example, is comparable in size to the absolute difference in tax preferences between the top and bottom income groups (0.24 points) or between the median and 80th percentile in terms of equality endorsement (0.19 points). The (negative) effect of the strong positive-sum group, at -0.3, is even greater. Moreover, there is little evidence of heterogeneous effects of the classes by income, education, political attention, or country.^10^

## Conclusion

We already know that in elite politics, those who hold different ideas about how the economy works advocate different prescriptions for taxing the rich. The same is true in the general population. Those who think that the economy is described in positive-sum terms are less supportive of progressive taxation. Those who think of the economy as an arena for distributive conflict advocate the most progressive tax structure. These patterns hold independently of material interests and egalitarian values. The central argument of this paper, then, is to highlight the place of descriptive understandings of the economy in characterising the kinds of people who support taxes on the rich.

Two clear limits to this argument need to be reiterated. First, zero-sum versus positive-sum economic thinking, and non-engagement, are not the only ways to look at public beliefs. Zero-sum characteristics have been highlighted (by economists and in economic psychology) as systematic features of lay economic thought with direct implications for tax policy preferences (Johnson, 2018; Rubin, 2003), while non-engagement emerges in the measurement of any other type of thinking, and also has a theoretical link to tax preferences As such these are obvious places to start. But other types of descriptive economic thinking might be more useful characterisations with reference to specific policy outcomes. We noted the cross-cutting question of empirical beliefs about inequality and mobility, but there are also other models of the national economy which might be more applicable to specific tax reform proposals (Ecnmy, 2017).

Second, this analysis describes, rather than explains, progressivity preferences. There are systematic differences in progressivity preferences across the identified groupings of economic thinking, but it is beyond the scope of this paper to adjudicate between various plausible causal directions that might account for the relationship. A consonant logic between economic thinking and progressivity preferences is equally consistent with the idea that understandings lead to preferences, or the reverse. Systematic differences may equally be due to some other feature of the way both are generated, such as the simultaneous exposure to both policy position and economic description, whether in political rhetoric or business news reporting. Experimental manipulations of the purported causes in these latter accounts are feasible and represent a next step for research. This paper ascertains that there is in fact an empirical regularity in need of explanation.

This paper also provides a descriptive account of zero-sum, positive-sum, and non-engaged economic thinking in the population across five post-industrial democracies. Independent of the direct link to progressivity preferences, this yields some notable findings. First, consistent zero-sum thinking was not to be found among the profiles of responses. Public thinking about economics is characterised by concern for distributive questions, especially (in the two largest groups) when considering what economics and economic policy are about. But otherwise, a recognition of positive-sum economics characterised all the engaged response profiles. On the other hand, fears of a hegemonic efficiency agenda, where questions of distribution are excised from economic thought, are equally misplaced: the strong positive-sum group represents only 7 per cent of the population. However, this group shares characteristics (high income and education, high attention to politics, older, male) with precisely those people most likely to be in positions to claim economic expertise and to influence policy. Elite ideational politics may have spread as far as this demographically similar group in the population, even as the rest of the democratic citizenry in all five countries studied here remain more enthusiastic about taxing the rich at high relative rates.

## Supplementary Material

Supplemental Appendix

## Data Availability

Supplementary material and data and code for replication are deposited at the Open Science Foundation osf.io at https://osf.io/u8gx2. The DOI is 10.17605/OSF.IO/U8GX2.

## Notes

1 Further details at https://www.respondi.com/EN/access-panel.

2 This survey was in the field during or just after the first wave of the Coronavirus pandemic, limiting strong claims about long-run representativeness. Nevertheless, it is not obvious that the pandemic would strongly change ways of thinking about the economy in the terms we are interested in, nor alter their relationships with tax policy preferences.

3 Full question texts and descriptive statistics for the variables used in these analyses can be found in the supplementary material.

4 Further details on these alternative specifications can be found in the supplementary material.

5 Using the AIC as the selection criterion is plausibly likely to yield too many latent classes to structure the data, in the sense that the latent class methodology attempts to attribute all possible variation in the data to the classes.

6 As a complement, and to consider the distinctiveness of the highly attentive: among those in the top 10 per cent of (reported) political attentiveness, the strong positive sum group is extremely over-represented, relative to the share of this group in the population as a whole (16 per cent among the attentive, versus 7 in the full sample).

7 Full statistics on country-specific population shares, and further country-specific analysis and discussion can be found in the supplementary information.

8 For the full text of the question, and descriptive statistics, see the supplementary material.

9 A full results table can be found in the supplementary material.

10 See supplementary material for full results.
