# Supplementary material for: Taxing the rich: public preferences and public understanding
Source: J Eur Public Policy. 2021 Dec 13;29(5):787–804. doi: 10.1080/13501763.2021.1992485 (PMC8966480; doi:10.1080/13501763.2021.1992485)
Supplement: Supplemental Appendix [file RJPP_A_1992485_SM9589.docx]

Supplementary Material for Taxing the Rich: Public Preferences and Public Understanding

Lucy Barnes

15th May 2021

# Data Deposit

This supplementary material file, and data and code for replication are deposited at the Open Science Foundation osf.io at <https://osf.io/u8gx2>. The DOI is 10.17605/OSF.IO/U8GX2.

# Survey Question Texts

## Economic Thinking Battery:

To what extent do you agree with the following statements about how the economy works:

|  |  | a. strongly disagree | b. disagree | c. neither agree nor disagree | d.  agree | e. strongly agree | Don’t know |
| --- | --- | --- | --- | --- | --- | --- | --- |
| Zero sum: conflict | If someone gets richer, somebody else gets poorer |  |  |  |  |  |  |
| Zero sum: purpose | For every new person that starts working, there is one less job for the existing workforce |  |  |  |  |  |  |
| Zero sum: labour market | Economics and economic policy are about distributing existing resources |  |  |  |  |  |  |
| Positive sum: conflict | If someone gets richer, it means that total wealth increases |  |  |  |  |  |  |
| Positive sum: purpose | New workers increase demand for goods and services and so create jobs for others |  |  |  |  |  |  |
| Positive sum: labour market | Economics and economic policy are about increasing the resources available |  |  |  |  |  |  |

## Additional Values, Attitudes, Behaviours Items:

### Values: Endorsement of the Necessity of Equality for Fairness

How far do you agree with the following statements about fairness taken generally? In each case, where would you place your opinion on this scale? 1 means you agree completely with the statement on the left; 10 means you agree completely with the statement on the right; and if your views fall somewhere in between, you can choose any number in between.

| 1 | 2 | 3 | 4 | 5 | 6 | 7 | 8 | 9 | 10 | Don’t know |
| --- | --- | --- | --- | --- | --- | --- | --- | --- | --- | --- |
| Fairness does not require attention to equality |  |  |  |  |  |  |  |  | Other things equal, fairness requires people get an equal share |  |

### Social Trust

Generally speaking, would you say that most people can be trusted, or that you can't be too careful in dealing with people? Please tell me on a score of 0 to 10, where 0 means you can't be too careful and 10 means that most people can be trusted.

| 0 | 1 | 2 | 3 | 4 | 5 | 6 | 7 | 8 | 9 | 10 | Don't know |
| --- | --- | --- | --- | --- | --- | --- | --- | --- | --- | --- | --- |
| You can’t be too careful |  |  |  |  |  |  |  |  |  | Most people can be trusted |  |

### Political Trust

Please tell me on a scale of 0-10 how much you personally trust the following groups. 0 means you do not trust them at all, and 10 means you have complete trust.

|  | 0 | 1 | 2 | 3 | 4 | 5 | 6 | 7 | 8 | 9 | 10 | Don’t know |
| --- | --- | --- | --- | --- | --- | --- | --- | --- | --- | --- | --- | --- |
| Politicians | Do not trust politicians at all |  |  |  |  |  |  |  |  |  | Have complete trust in politicians |  |

## Demographic Characteristics

### Gender

Are you…

1. Male
2. Female
3. Prefer not to say

Recoded to differentiate male from non-male identification.

### Age

What was your age last birthday? [write in numeric or "prefer not to say"]

### Education

What is the highest educational level that you have attained? [NOTE: if you are a student, indicate the highest level you expect to complete]:

1. No formal education
2. Incomplete primary school
3. Complete primary school
4. Incomplete secondary school: technical/vocational type
5. Complete secondary school: technical/vocational type
6. Incomplete secondary: university-preparatory type
7. Complete secondary: university-preparatory type
8. Some university-level education, without degree
9. University-level education, with degree
10. Don’t know

Recoded to four categories (plus don't know): a-d, f: less than secondary; e, secondary vocational; g-h, secondary academic; i, degree.

### Labour force status

Which of these descriptions applies to your main activity in the last 7 days?

1. in paid work (or away temporarily) (employee, self-employed, working for your family business)
2. in education, (not paid for by employer) even if on vacation
3. unemployed and actively looking for a job
4. unemployed, wanting a job but not actively looking for a job
5. permanently sick or disabled
6. retired
7. in community or military service
8. doing housework, looking after children or other persons
9. other
10. Don’t know

### Employment type

The next three questions are about your experiences in paid work. Please answer with reference to your main job, if you are currently working. If you are not working at the moment, think about your most recent job.

Are/were you:

1. an employee
2. self-employed
3. working for your own family business?
4. Never had a paid job
5. Don’t know

### Income

Income levels are asked in country-specific questions in which the levels provided were calibrated to generate 15 categories with cutpoints at the same quantile of the income distribution as those for the UK, values for which were taken from the British Election Study. These mappings were made using the World Income Inequality database at https://wid.world/simulator/.

Into which of these categories does your total household income before tax fit? Think about income from all sources – including benefits, savings and so on?

UK:

|  | Annual | Monthly | Weekly |
| --- | --- | --- | --- |
| 1 | Under £2,600 | Under £217 | Under £50 |
| 2 | £2,600 - £5,199 | £217 - £434 | £50 - £99 |
| 3 | £5,200 - £10,399 | £435 - £866 | £100 - £199 |
| 4 | £10,400 - £15,599 | £867 - £1,299 | £200 - £299 |
| 5 | £15,600 - £20,799 | £1,300 - £1,732 | £300 - £399 |
| 6 | £20,800 - £25,999 | £1,733 - £2,166 | £400 - £499 |
| 7 | £26,000 - £31,199 | £2,167 - £2,599 | £500 - £599 |
| 8 | £31,200 - £36,399 | £2,600 - £3,032 | £600 - £699 |
| 9 | £36,400 - £39,999 | £3,033 - £3,333 | £700 - £769 |
| 10 | £40,000 - £44,999 | £3,334 - £3,749 | £770 - £865 |
| 11 | £45,000 - £49,999 | £3,750 - £4,166 | £866 - £961 |
| 12 | £50,000 - £59,999 | £4,167 - £4,999 | £962 - £1,153 |
| 13 | £60,000 - £74,999 | £5,000 - £6,249 | £1,154 - £1,442 |
| 14 | £75,000 - £99,999 | £6,250 - £8,333 | £1,443 - £1,923 |
| 15 | £100,000 or more | £8,333 or more | £1,924 or more |

Don’t know

DK:

|  | Annual | Monthly | Weekly |
| --- | --- | --- | --- |
| 1 | under 51.144 kr. | under 4.262 kr. | under 983 kr. |
| 2 | 51.145 kr. - 77.304 kr. | 4.262 kr. - 6.442 kr. | 983 kr. - 1.486 kr. |
| 3 | 77.305 kr. - 117.624 kr. | 6.443 kr - 9.802 kr. | 1.487 kr. - 2.262 kr. |
| 4 | 117.625 kr. - 156.624 kr. | 9.803 kr. - 13.052 kr. | 2.263 kr. - 3.012 kr. |
| 5 | 156.625 kr. - 210.600 kr. | 13.053 kr. - 17.550 | 3.013 kr. - 4.050 kr. |
| 6 | 210.601 kr. - 255.288 kr. | 17.551 kr - 21.274 kr. | 4.051 kr. - 4.909 kr. |
| 7 | 255.289 kr. - 293.364 kr. | 21.275 kr. - 24.447 kr. | 4910 kr. - 5.641 kr. |
| 8 | 293.365 kr. - 328.992 kr. | 24.448 kr - 27.416 kr. | 5.642 kr. - 6.326 kr. |
| 9 | 328.993 kr. - 348.504 kr. | 27.417 kr - 29.042 kr. | 6.327 kr. - 6.702 kr. |
| 10 | 348.505 kr. - 384.732 kr. | 29.043 kr - 32.061 kr. | 5.703 kr. - 7.398 kr. |
| 11 | 384.733 kr. - 408.684 kr. | 32.062 kr. - 34.057 kr. | 7.399 kr. - 7.859 kr. |
| 12 | 408.685 kr. - 488.376 kr. | 34.058 kr. - 40.698 kr. | 7.860 kr. - 9.391 kr. |
| 13 | 488.377 kr. - 575.628 kr. | 40.699 kr. - 47.969 kr. | 9.392 kr. - 11.069 kr. |
| 14 | 575.629 kr. - 797.268 kr. | 47.970 kr. - 66.439 kr. | 11.070 kr. - 15.332 kr. |
| 15 | 797.269 kr. or more | 66.440 kr. Or more | 15.333 kr. or higher |

Don’t know

FR:

|  | Annual | Monthly | Weekly |
| --- | --- | --- | --- |
| 1 | under €3.780 | under €315 | under €73 |
| 2 | €3.781 - €5748 | €315 - €479 | €73 - €111 |
| 3 | €5.749 - €12.084 | €480 - €1.007 | €112 - €232 |
| 4 | €12.085 - €17.376 | €1.008 - €1.448 | €233 - €334 |
| 5 | €17.377 - €23.196 | €1.449 - €1.933 | €335 - €446 |
| 6 | €23.197 - €27.852 | €1.934 - €2.321 | €447 - €536 |
| 7 | €27.853 - €32.760 | €2.322 - €2.730 | €537 - €630 |
| 8 | €32.761 - €39.132 | €2.731 - €3.261 | €631 - €753 |
| 9 | €39.133 - €42.048 | €3.262 - €3.504 | €754 - €809 |
| 10 | €42.049 - €46.392 | €3.505 - €3.866 | €810 - €892 |
| 11 | €46.393 - €48.936 | €3.867 - €4.078 | €893 - €941 |
| 12 | €48.937 - €60.432 | €4.079 - €5.036 | €942 - €1.162 |
| 13 | €60.433 - €71.280 | €5.037 - €5.940 | €1.163 - €1.371 |
| 14 | €71.281 - €99.348 | €5.941 - €8.279 | €1.372 - €1.911 |
| 15 | €99.349 or more | €8.280 or more | €1.912 or more |

Don’t know

GER:

|  | Annual | Monthly | Weekly |
| --- | --- | --- | --- |
| 1 | unter €3.780 | unter €315 | unter €73 |
| 2 | €3.781 - €5748 | €315 - €479 | €73 - €111 |
| 3 | €5.749 - €12.084 | €480 - €1.007 | €112 - €232 |
| 4 | €12.085 - €18.036 | €1.008 - €1.503 | €233 - €347 |
| 5 | €18.037 - €24.300 | €1.504 - €2.025 | €348 - €467 |
| 6 | €24.301 - €31.260 | €2.026 - €2.605 | €468 - €601 |
| 7 | €31.261 - €38.004 | €2.606 - €3.167 | €602 - €731 |
| 8 | €38.005 - €45.108 | €3.168 - €3.759 | €732 - €867 |
| 9 | €45.109 - €49.644 | €3.760 - €4.137 | €868 - €955 |
| 10 | €49.645 - €58.200 | €4.138 - €4.850 | €956 - €1.119 |
| 11 | €58.201 - €62.220 | €4.851 - €5.185 | €1.120 - €1.197 |
| 12 | €62.221 - €78.060 | €5.186 - €6.505 | €1.198 - €1.501 |
| 13 | €78.061 - €94.164 | €6.505 - €7.847 | €1.502 - €1.811 |
| 14 | €94.165 - €131.160 | €7.848 - €10.930 | €1.812 - €2.522 |
| 15 | €131.161 oder mehr | €10.931 oder mehr | €2.523 oder mehr |

Don’t know

US:

|  | Annual | Monthly | Weekly |
| --- | --- | --- | --- |
| 1 | under $2000 | unter $167 | unter $38 |
| 2 | $2000-$3999 | $168 - $333 | $39 - $77 |
| 3 | $4000 - $8552 | $334 - $713 | $78 - $164 |
| 4 | $8,533 - $17,976 | $712 - $1,498 | $165 - $346 |
| 5 | $17,977 - $30,096 | $1,499 - $2,508 | $347 - $579 |
| 6 | $30,097 - $43,560 | $2,509 - $3,630 | $580 - $838 |
| 7 | $43,561 - $56,184 | $3,631 - $4,682 | $839 - $1,080 |
| 8 | $56,185 - $69,648 | $4,683 - $5,804 | $1,081 - $1,339 |
| 9 | $69,649 - $76,836 | $5,805 - $6,403 | $1,340 - $1,478 |
| 10 | $76,837 - $90,288 | $6,404 - $7,524 | $1,479 - $1,736 |
| 11 | $90,289 - $99,492 | $7,525 - $8,291 | $1,737 - $1,913 |
| 12 | $99,493 - $131,256 | $8,292 - $10,938 | $1,913 - $2,524 |
| 13 | $131,257 - $164,700 | $10,939 - $13,725 | $2,525 - $3,167 |
| 14 | $164,701 - $250,812 | $13,726 - $20,901 | $3,168 - $4,823 |
| 15 | $250,813 or higher | $20,902 or higher | $4,824 or more |

Don’t know

The fifteen categories for each country are coded into five groups as follows:

1: 1-4; 2: 5-6; 3: 7-9; 4: 10-12; 5: 13-15.

The aim of these groupings is to create relatively equal-sized income groups within the sample.

# Descriptive Statistics

|  | | | |
| --- | --- | --- | --- |
| Categorical variables | | | |
|  | N | Sample share |  |
| Non-male | 5278 | 0.50 |  |
| Denmark | 5278 | 0.20 |  |
| France | 5278 | 0.20 |  |
| Germany | 5278 | 0.20 |  |
| UK | 5278 | 0.20 |  |
| USA | 5278 | 0.20 |  |
| Lowest income | 4887 | 0.18 |  |
| Income 2 | 4887 | 0.19 |  |
| Income 3 | 4887 | 0.24 |  |
| Income 4 | 4887 | 0.20 |  |
| Highest income | 4887 | 0.19 |  |
| Less than secondary | 5246 | 0.10 |  |
| Secondary vocational | 5246 | 0.27 |  |
| Secondary academic | 5246 | 0.22 |  |
| Degree | 5246 | 0.41 |  |
| Continuous variables | | | |
|  | N | Mean | SD |
| Age | 5261 | 47.30 | 15.96 |
| Social trust | 5148 | 5.14 | 2.65 |
| Trust politicians | 5139 | 4.03 | 2.70 |
| Values equality | 4831 | 6.90 | 2.42 |
| Summary of tax and thinking items as-if continuous | | | |
| Progressivity | 5027 | 3.90 | 0.79 |
| Zero sum: conflict | 5043 | 3.14 | 1.21 |
| Zero sum: purpose | 4858 | 3.39 | 1.02 |
| Zero sum: labour market | 4929 | 2.66 | 1.07 |
| Positive sum: conflict | 4920 | 3.01 | 1.12 |
| Positive sum: purpose | 4801 | 3.53 | 0.89 |
| Positive sum: labour market | 5000 | 3.76 | 0.87 |
|  |  |  |  |

Table A1: Descriptive Statistics

# Latent Class Model Selection

Selecting the number of classes for the latent variable model is a judgment call based on both the statistical fit of the model and the interpretability of the latent classes which are generated by the model fit. Larger numbers of classes, especially in models with covariates, are also much more computationally intensive.

Figure A1 and Table A2 below show fit statistics for a number of combinations of number of classes and covariate combinations in the models. The plateauing of improvement to the AIC and in particular the worsening of fit by the BIC, as well as a worsening interpretability of classes beyond 7 (with covariates) led me to select that model.

As indicated in Table A2, however, marginal improvements in the null (no covariate) LCA model continue through nine classes (at 10, the BIC increases). Moreover this solution identifies interpretable classes which correspond to those obtained in the covariate model with seven classes, in terms of the conditional response profiles of the classes. Thus in comparing LCA and my preferred LCR (covariates for age, gender, income, country and education) models, I refer to the nine-class fit.

|  | LCA (no covariates) | | LCR (covariates for age, gender, education, country) | | LCR (covariates for age, gender, education, country, income) | |
| --- | --- | --- | --- | --- | --- | --- |
|  | AIC | BIC | AIC | BIC | AIC | BIC |
| 1 | 98193 | 98390 | 97252 | 97448 |  |  |
| 2 | 93366 | 93766 | 92931 | 92856 |  |  |
| 3 | 90348 | 90952 | 89318 | 90053 |  |  |
| 4 | 89098 | 89906 | 87984 | 88989 | 81768 | 92838 |
| 5 | 88335 | 89347 | 87122 | 88396 | 81009 | 82372 |
| 6 | 87701 | 88918 | 86431 | 87973 | 80286 | 81941 |
| 7 | 87277 | 88696 | 85905 | 87717 | 79903 | 81849 |
| 8 | 86907 | 88531 | 85959 | 88039 |  |  |
| 9 | 86545 | 88372 |  |  |  |  |
| 10 | 86435 | 88466 |  |  |  |  |

Table A2: Latent class model fit (Information Criteria) across different covariate specifications.

# Latent Class Models with or without Covariates

As discussed in the main text, I prefer to estimate the link between covariates and latent classes in a one-step model due to the tendency for separate estimation (first fitting classes, then separately estimating relationships to covariates) tends to lead to bias in estimating the relationships. However, in terms of the classes recovered, the substantive results are relatively similar if we adopt a latent class model without covariates.

Figures A2 to A4 show the conditional response probability profile heatmaps for seven, eight, and nine-class models estimated without covariates.

Figure A2: Conditional response probability profile heatmap, seven latent classes, no covariates.

In this 7-class model we can readily identify the don't know, non-committal, and acquiescing groups as usual (with 5.9, 6.7, and 10.5 percent population shares, respectively -- not dissimilar to the shares from the 7-class model with covariates presented in the main text). The strong positive sum group is also clearly identifiable, but much larger in this estimation at 12.1% of the population. The three groups that remain consist of one small class (3.1% population share) characterised by disagreement with the statements provided, except for the strong endorsement of the idea of zero-sum conflict between rich and poor. This group also disagrees with the positive sum statement about the fate of the rich very strongly. These groupings comprise only around 40% of the population, leaving the majority of the population split between the two largest groups. The smaller of these two (with 27.6% of the population) is slightly more oriented towards class conflict than the larger, while the latter more strongly rejects zero-sum labour market ideas (and endorses positive sum labour market ideas), and has responses concentrated on agreement with both of the two "purpose of the economy" statements. These groupings may be read as broad center-left, and broad center-right, but their overall size in the population, and the lack of clarity in the response patterns in differentiating the two (as well as the statistical diagnosis that the fit of the model is improved by the addition of additional classes) indicates that this version of reducing the data is not the most useful.

Figure A3: Conditional response probability profile heatmap, eight latent classes, no covariates.

Adding an additional class, to create an eight-class model, yields more familiar patterns among the larger classes, and a clearer differentiation between the response profiles among them. Specifically, the strong positive sum group is smaller, a weak positive sum pattern re-emerges as the modal class, and the two remaining large classes comprise the left-neoliberal pattern (labour market positive-sum orientations plus a commitment to both purposes and some endorsement of zero-sum conflict) and the class conflict group (clear endorsement of the negative-sum conflict idea, and disagreement with the positive-sum view of rich-other conflict). The three non-response profiles are also evident. The eighth class, comprising 7.6% of the population, has a profile which looks similar to the left-neoliberal pattern in terms of conflict with the rich and labour market orientations, but which has a particular tendency to respond "don't know" on the questions about what economics and economic policy are about. Relative to the seven-class model with covariates, then, we recover the same classes here, plus this one additional "no purpose" group. I do not dwell on these interpretations as we replicate them again as the fit of the model continues to improve with the addition of a further class.

Thus Figure RMXX shows the analogous heatmap for the nine-class model estimated without covariates. Here I have ordered the classes from most to least prevalent, and provided labels to the classes. In this figure we see the seven "original" classes from the covariate model, plus the two novel groupings -- "no purpose" and the disagreement-prone "rejection and class conflict" groups that the 7- and 8-class models without covariates identified.

Figure A4: Conditional response probability profile heatmap, nine latent classes, no covariates. Classes ordered by population share from largest (top) to smallest (bottom).

The primary difference between this mapping and the one identified by the seven-class covariate model is in the relative sizes of the groups. The weak positive sum group remains the most prevalent, comprising 26.7% of the population, but class conflict and left-neoliberal groups change place in the ordering, the former here comprising 20.2% of the population and the latter 15.2%. Intuitively, this is due to the splitting out from the left-neoliberal grouping those who are here moved into the "no purpose" class, and the grouping of more moderate profiles within the class-conflict group as the variance-minimizing solution once the more extreme "rejection and conflict" profiles are separated into a different group.

Thus, overall the underlying latent classes remains very similar when incorporating or when excluding covariates from the analyses. As such, the latent class measurement in the covariate model does not seem to be driven by the particular specification of the structural component.

Moreover, separating estimation into two steps has been shown to bias estimates of the relationships between covariates and class membership, and specifically to underestimate the associations (Bolck, Croon & Hagenaars, 2004). Figure A5 plots the coefficients from multinomial logit models of class membership estimates in the nine-class LCA (no covariate) model alongside those from the simultaneous estimation LCR, for those classes with the same substantive interpretations, which illustrates that this is generally also the case in this application. Although the differences in coefficient estimates are rarely statistically significant, where they are it is due to stronger effects estimated in the LCR, in particular for the impact of income on the strong positive sum class.

Figure A5: Coefficients on covariates in multinomial models of class categorisation. Estimated in one-step (blue) and three-step (red) latent class models. Omitted category is Acquiescer.

The lack of huge differences here, implies that the choice between the covariate-LCR and null-LCA matters relatively little for the descriptive characterisation of the classes. Figure A6 shows the mean values of income, education, political attention and age in each of the seven overlapping classes, relative to the sample means for these variables. Both the positive-sum groups, and in particular the strong positive sum group, are richer, better educated, more politically attentive, and older than average. The non-committal and don't know groups are systematically younger, poorer, less attentive and have less education. The remaining three groups -- the left neoliberals and class conflict groups, and aqcuiescers, are close to the sample averages, with the odd exception of the acquiescer group reporting high levels of political attention.

Figure A6: Characterisation of economic thinking classes by selected covariates. Height of bars indicates class mean value relative to the overall sample mean, with higher values indicating higher income, education, political attention levels and higher ages.

(a) Classes from 7-class LCR model with age, gender, education, income and country covariates.

(b) Classes from 9-class LCA model – the seven analogous classes to those recovered in the 7-class LCR

# Characterising the Latent Classes: Covariate Impact on Priors

There are two ways to think about characterising the different classes of economic thinking revealed in the population. The main manuscript provides descriptive statistics on those assigned to each class -- i.e. on the basis of posterior probability.

Here we can think about the impact of covariates on the priors of the LCA model, characterising different population groups in terms of their likelihood of being in each class. These are the coefficients from the one-step model analogous to those from a multinomial regression with class membership as its outcome (as per section 5). Figure A7 shows these patterns for income and for education.^^[[1]](#footnote-1)^^ These predicted probabilities show the impact of these variables net of relationships with other covariates.

Figure A7. Relationships between covariates and prior probability of class membership. Estimates from seven class model with covariates: country, age, gender, education and income.

The figures show quite clear income and education gradients for the two largest groups, with weak positive-sum classification more likely among those with higher incomes and higher, academic education levels. The left neoliberal class is less common in the two highest income groups, but shows a weak inverted-U shape with regard to education. The don't know category is unsurprisingly more common at lower levels of education, while there is a very strong, non-linear link between membership of the highest income group and the strongly-positive sum class.

# Country-Specific Analyses and Country Comparisons

Figure A8 (overleaf) shows the results of estimating seven-class LCA models (without covariates) within each country separately. In general, classes with similar profiles emerge within each country – with three important exceptions, highlighted in red in the figure. Specifically, in the UK a class is created which is characterised by a high propensity to reply “don’t know” to the two questions about the purpose of economics and economic policy. On the other items this group tends to observe both zero-sum conflict between rich and poor, but positive sum labour market dynamics. This profile (on the conflict and labour market items) is generally associated with the left-neoliberals. Unsurprisingly, then, respondents classified in this novel British “no purpose” class under this national estimation are drawn (in a roughly 4:5 ratio) from the “don’t know” and “left neoliberal” classes as estimated using the full sample latent class model. Thus we do not see a left neoliberal class in the British data. The weak positive sum group is correspondingly large, absorbing the remainder of those classed as left-neoliberal in the full sample to estimation.

In Germany and France, a different novel profile emerges, which is one of rejection of all of the statements except for that describing the rich getting richer at the expense of others. These “rejectionist zero sum” groups consist almost entirely of those who are categorised in the “class conflict” group in the full sample estimation. They are also relatively few in number: 33 respondents in Germany and 20 in France (or 3.1 and 1.9 percent of each national sample, respectively).

In the German estimation this rejectionist class conflict group replaces the much larger general class conflict category. Under the full sample estimation this category draws population from across all the nationally estimated groups, but primarily from those classed as left neoliberals under the national estimation. In the French estimation there is a distinct class conflict group as well as the “rejectionist class conflict” class, but the national estimation does not identify any strong positive sum group. Those who are classified as strong positive sum in the full sample estimation are drawn from the weak positive sum group and the aqcuiescers, in the French national estimation – but it is important to note that there are very few of them: only 28 respondents.

Figure A8: Latent class profiles: seven class models estimated within countries

Tables A3 to A6 illustrate the population shares in each class, as estimated in the full sample and the national samples.

Note too that the additional profiles in the nationally-specific estimations – the “rejectionist class conflict” and “no purpose” groups – are those which emerge in the 9-class LCA that pools across all countries for its estimation, again indicating consistency across the categorisations.

Table A3: Population shares within each category, nationally-estimated classes (class population shares, no-covariate LCA)

|  | Denmark | USA | Germany | UK | France |
| --- | --- | --- | --- | --- | --- |
| Weak positive sum | 29.8 | 33.5 | 29.4 | 45.0 | 22.4 |
| Left neoliberal | 32.2 | 21.2 | 27.0 | (11.1) | 16.2 |
| Class conflict | 10.6 | 9.1 | (3.9) | 8.2 | 32.4 |
| Strong positive sum | 7.5 | 9.6 | 12.7 | 11.4 | (2.2) |
| Non-committal | 8.9 | 14.0 | 8.0 | 9.8 | 8.2 |
| Acquiescers | 5.7 | 7.5 | 14.7 | 10.3 | 9.8 |
| High don't know | 5.3 | 5.0 | 4.3 | 4.2 | 8.9 |

Table A4: Population shares within each category, full sample-estimated classes (class population shares, covariate LCR)

|  | Denmark | USA | Germany | UK | France |
| --- | --- | --- | --- | --- | --- |
| Weak positive sum | 41.4 | 24.3 | 23.9 | 28.9 | 25.6 |
| Left neoliberal | 25.4 | 25.8 | 22.1 | 27.7 | 18.6 |
| Class conflict | 4.3 | 11.4 | 22.0 | 11.7 | 28.9 |
| Strong positive sum | 12.8 | 8.4 | 5.5 | 5.4 | 2.9 |
| Non-committal | 6.6 | 10.6 | 5.7 | 9.2 | 11.1 |
| Acquiescers | 6.1 | 14.4 | 16.2 | 9.7 | 3.5 |
| High don't know | 3.3 | 5.0 | 4.5 | 7.5 | 9.4 |

Table A5: Population shares within each category, nationally-estimated classes (predicted class, covariate LCR)

|  | Denmark | USA | Germany | UK | France |
| --- | --- | --- | --- | --- | --- |
| Weak positive sum | 30.1 | 33.9 | 32.0 | 47.0 | 24.4 |
| Left neoliberal | 32.0 | 21.4 | 26.7 | (10.1) | 15.2 |
| Class conflict | 9.5 | 8.2 | (3.2) | 7.6 | 32.7 |
| Strong positive sum | 7.5 | 9.4 | 11.5 | 10.2 | (2.2) |
| Non-committal | 9.5 | 14.3 | 8.1 | 10.1 | 8.4 |
| Acquiescers | 5.6 | 7.7 | 14.1 | 10.0 | 8.2 |
| High don't know | 5.3 | 5.2 | 4.4 | 4.3 | 9.0 |

Table A6: Population shares within each category, full sample-estimated classes (predicted class, covariate LCR)

|  | Denmark | USA | Germany | UK | France |
| --- | --- | --- | --- | --- | --- |
| Weak positive sum | 43.4 | 25.2 | 23.9 | 30.3 | 26.1 |
| Left neoliberal | 25.6 | 27.6 | 23.6 | 28.6 | 17.6 |
| Class conflict | 3.4 | 9.6 | 20.8 | 10.2 | 29.2 |
| Strong positive sum | 12.6 | 8.1 | 5.8 | 4.9 | 2.9 |
| Non-committal | 6.4 | 13.7 | 5.7 | 9.5 | 11.5 |
| Acquiescers | 5.6 | 13.7 | 15.5 | 9.5 | 3.1 |
| High don't know | 3.1 | 5.0 | 4.8 | 7.7 | 9.7 |

Clearly this is not a "hard test" for the economic thinking classes, given that these nationally-specific patterns combined are what drove the original classifications and interpretations. Nevertheless, I think this does provide validation at least to the extent that it is possible to get it from within-sample analyses.

However, there is a sense in which it makes more sense to create the classes -- the archetypes of thinking, if you will -- from the data as a whole, and then to ask whether there are categories within this "universal" set which are poorly populated in particular countries. This also takes the cross-national variation seriously, but with reference to the generally-derived classes.

Beyond its use for comparisons to the nationally-specific estimations, Table RMXXb is particularly useful in thinking about the cross-national nature of the data in more depth. First, it highlights the consistency of the left-neoliberal population share across the five countries (with the partial exception of France, which has a smaller share in this class). In contrast, the class conflict class is very different in size across the different countries, much larger in the continental European countries, smaller in Denmark, and with the two Anglophone nations in the middle.

Denmark is unique in the high share positive sum profiles, with disproportionately large population shares in both the weak positive sum and strong positive sum groups, relative to the other countries. While this might seem like a surprising result in the context of the stereotypical social democratic Scandinavian welfare state, in fact it is consistent with high degree of economic liberalism in Danish economic thought and policy (XX citations XX). Perhaps more speculatively, it is also plausible that *in fact* doing better at ensuring that economic growth is equitably shared is reflected in the absence of expressions of class conflict in Denmark, and the greater share of people expressing positive sum thinking. Denmark is also distinctive in the relatively small share occupying the three types of non-response class.

Across the other countries, non-response also follows slightly distinctive patterns, with the French and Americans more likely to have non-committal response profiles, and the Germans joining the Americans with high shares of the acquiescence class. The share of acquiesce profiles in France is outlyingly low, but compensated in terms of overall non-response profiles by a high share in the “don’t know” class.

In all countries the explicit “don’t know’ non-response profile is the smallest of the three non-informative classes. This is important in terms of the payoffs from the latent class modelling. On a variable-by-variable approach, a considerable amount of noise would be imported into the analysis by reading individual responses from the acquiescing and non-committal classes as providing the same information as substantive agreement, or unique item indifference (neither-nor responses).

# The Politically Attentive

Are the politically attentive different, in their distribution across classes of economic thinking, than the general population? I noted in the main text that among those in the top 10% of (reported) political attentiveness, the strong positive sum group is extremely over-represented, relative to the share of this group in the population as a whole (16% among the attentive, versus 7 in the full sample). Here I provide some further details on that group. Notably, acquiescers appear among the (reportedly) attentive at an equally disproportionate level (21% vs 10 in the full sample). The class conflict and weak positive sum groups are found among the attentive in similar proportions as in the general population, while the left-neoliberal group, as well as the don't know and non-committal groups, are under-represented.

| **Group** | **N in high attention** | **% high attention** | **% full sample** |
| --- | --- | --- | --- |
| Acquiescer | 113 | 21.4 | 10 |
| Class conflict | 94 | 17.8 | 15.8 |
| Don't know | 11 | 2.1 | 6 |
| Left neoliberal | 76 | 14.4 | 23.9 |
| Non-committal | 20 | 3.8 | 8.7 |
| Strong + | 84 | 15.9 | 6.9 |
| Weak + | 129 | 24.5 | 28.6 |

Table A7: the distribution of the highly politically attentive across classes.

# Main Regression Analyses: Full Table

| Table A9: Regression output for models displayed in figure 2 in the main paper. Baseline (omitted) categories for categorical covariates are: weak positive sum (economic thinking), Denmark (country), lowest quintile (income), incomplete secondary (education) and don't know (party vote). | | | | | | | |
| --- | --- | --- | --- | --- | --- | --- | --- |
|  | | | | | | | |
|  | **Support for progressive tax structure** | | | | | | |
|  | **Model 1** | **Model 2** | **Model 3** | **Model 4** | **Model 5** | **Model 6** | **Model 7** |
|  | | | | | | | |
| Class conflict | 0.397^***^ | 0.359^***^ | 0.412^***^ | 0.395^***^ | 0.319^***^ | 0.212^***^ | 0.155^***^ |
|  | (0.045) | (0.047) | (0.047) | (0.047) | (0.048) | (0.049) | (0.048) |
| Left neolib | 0.040 | 0.011 | 0.083^*^ | 0.073 | 0.011 | 0.015 | -0.006 |
|  | (0.043) | (0.043) | (0.044) | (0.045) | (0.045) | (0.045) | (0.044) |
| Strong + | -0.377^***^ | -0.382^***^ | -0.344^***^ | -0.336^***^ | -0.278^***^ | -0.297^***^ | -0.249^***^ |
|  | (0.058) | (0.058) | (0.057) | (0.057) | (0.057) | (0.057) | (0.056) |
| Don't know | 0.332^***^ | 0.341^***^ | 0.314^***^ | 0.333^***^ | 0.382^***^ | 0.356^***^ | 0.357^***^ |
|  | (0.074) | (0.074) | (0.073) | (0.073) | (0.086) | (0.087) | (0.086) |
| Non-committal | -0.195^***^ | -0.230^***^ | -0.137^**^ | -0.148^***^ | -0.173^***^ | -0.171^***^ | -0.198^***^ |
|  | (0.054) | (0.054) | (0.054) | (0.055) | (0.056) | (0.055) | (0.055) |
| Acquiescer | 0.459^***^ | 0.434^***^ | 0.429^***^ | 0.447^***^ | 0.329^***^ | 0.345^***^ | 0.364^***^ |
|  | (0.061) | (0.062) | (0.061) | (0.061) | (0.062) | (0.063) | (0.062) |
| France |  | 0.105^***^ | 0.096^**^ | 0.059 | 0.062 | 0.010 | 0.071^*^ |
|  |  | (0.038) | (0.038) | (0.040) | (0.041) | (0.041) | (0.041) |
| Germany |  | 0.167^***^ | 0.154^***^ | 0.115^***^ | 0.119^***^ | 0.089^**^ | 0.117^***^ |
|  |  | (0.037) | (0.037) | (0.039) | (0.039) | (0.039) | (0.040) |
| UK |  | 0.158^***^ | 0.158^***^ | 0.119^***^ | 0.090^**^ | 0.032 | 0.118^***^ |
|  |  | (0.037) | (0.037) | (0.038) | (0.038) | (0.038) | (0.041) |
| USA |  | 0.239^***^ | 0.233^***^ | 0.206^***^ | 0.206^***^ | 0.132^***^ | 0.199^***^ |
|  |  | (0.037) | (0.037) | (0.039) | (0.039) | (0.039) | (0.042) |
| Political attention |  |  |  |  |  | 0.018^***^ | 0.017^***^ |
|  |  |  |  |  |  | (0.005) | (0.005) |
| Age |  |  | 0.008^***^ | 0.009^***^ | 0.009^***^ | 0.008^***^ | 0.009^***^ |
|  |  |  | (0.001) | (0.001) | (0.001) | (0.001) | (0.001) |
| Income 2 |  |  |  | 0.007 | 0.026 | 0.030 | 0.022 |
|  |  |  |  | (0.037) | (0.038) | (0.037) | (0.037) |
| Income 3 |  |  |  | -0.002 | 0.004 | 0.004 | 0.011 |
|  |  |  |  | (0.037) | (0.037) | (0.037) | (0.036) |
| Income 4 |  |  |  | -0.094^**^ | -0.075^*^ | -0.067^*^ | -0.057 |
|  |  |  |  | (0.039) | (0.040) | (0.039) | (0.039) |
| Income 5 |  |  |  | -0.121^***^ | -0.093^**^ | -0.075^*^ | -0.055 |
|  |  |  |  | (0.043) | (0.043) | (0.043) | (0.042) |
| Secondary vocational |  |  |  | 0.136^***^ | 0.149^***^ | 0.144^***^ | 0.147^***^ |
|  |  |  |  | (0.044) | (0.044) | (0.044) | (0.043) |
| Secondary academic |  |  |  | 0.132^***^ | 0.149^***^ | 0.156^***^ | 0.147^***^ |
|  |  |  |  | (0.045) | (0.045) | (0.045) | (0.044) |
| Degree |  |  |  | 0.168^***^ | 0.191^***^ | 0.194^***^ | 0.167^***^ |
|  |  |  |  | (0.043) | (0.043) | (0.043) | (0.043) |
| Values: equality |  |  |  |  | 0.066^***^ | 0.071^***^ | 0.058^***^ |
|  |  |  |  |  | (0.005) | (0.005) | (0.005) |
| Party: far left |  |  |  |  |  |  | 0.202^***^ |
|  |  |  |  |  |  |  | (0.060) |
| Party: far right |  |  |  |  |  |  | -0.173^***^ |
|  |  |  |  |  |  |  | (0.059) |
| Party: green |  |  |  |  |  |  | 0.108^*^ |
|  |  |  |  |  |  |  | (0.059) |
| Party: left |  |  |  |  |  |  | 0.098^*^ |
|  |  |  |  |  |  |  | (0.051) |
| Party: liberal/centre |  |  |  |  |  |  | -0.158^***^ |
|  |  |  |  |  |  |  | (0.057) |
| Party: other |  |  |  |  |  |  | -0.063 |
|  |  |  |  |  |  |  | (0.063) |
| Party: right |  |  |  |  |  |  | -0.191^***^ |
|  |  |  |  |  |  |  | (0.051) |
| Party: regional |  |  |  |  |  |  | -0.137 |
|  |  |  |  |  |  |  | (0.099) |
| Additional controls: gender, age, labour force status, employment type, social and political trust |  |  |  |  |  | Y | Y |
| N | 4665 | 4665 | 4665 | 4665 | 4410 | 4393 | 4393 |
| R-squared | 0.047 | 0.057 | 0.090 | 0.096 | 0.135 | 0.156 | 0.184 |
| Adj. R-squared | 0.046 | 0.055 | 0.085 | 0.089 | 0.129 | 0.149 | 0.176 |
|  | | | | | | | |
| ^***^p < .01; ^**^p < .05; ^*^p < .1 | | | | | | | |

# Additional Analyses: Heterogeneous Effects

Table A10 considers whether the links between the latent classes and tax progressivity outcomes are specifically driven by particular subgroups in the data -- for example, the highly politically attentive. The table shows the results of models including interactions between the latent classes and selected covariates. There is really no evidence that this is the case.

Figure A9 illustrates the null interaction results (from the table) translated across into predicted probabilities, considering the predicted tax progressivity positions of the four "substantive" classes at median and maximum levels of political attention.

Figure A9: Preferred progressivity levels by economic thinking class and political attention. Estimates based on Model 1, Table A10.

| Table A10: Heterogeneous effects? Interactions between latent class and political attention, income, education and country. | | | | |
| --- | --- | --- | --- | --- |
|  | **Tax progressivity** | | | |
|  | **Model 1** | **Model 2** | **Model 3** | **Model 4** |
|  | | | | |
| Class conflict | 0.028 | 0.212^*^ | 0.173 | 0.447^**^ |
|  | (0.130) | (0.111) | (0.145) | (0.180) |
| Left neolib | 0.026 | 0.043 | -0.010 | 0.003 |
|  | (0.136) | (0.110) | (0.134) | (0.091) |
| Strong + | 0.033 | -0.238 | -0.507^***^ | -0.450^***^ |
|  | (0.216) | (0.173) | (0.192) | (0.096) |
| Don’t know | 0.135 | 0.450^**^ | 0.584^**^ | 0.291 |
|  | (0.194) | (0.196) | (0.232) | (0.230) |
| Non-commit | -0.012 | -0.038 | -0.233 | -0.289^**^ |
|  | (0.146) | (0.132) | (0.162) | (0.132) |
| Acquiescer | 0.085 | 0.271^*^ | 0.422^**^ | 0.382^**^ |
|  | (0.182) | (0.142) | (0.174) | (0.174) |
| Pol. attention | 0.016 |  |  |  |
|  | (0.012) |  |  |  |
| Income (continuous, 1-5) |  | -0.009 |  |  |
|  |  | (0.020) |  |  |
| Education (continuous, 1-4) |  |  | 0.059^**^ |  |
|  |  |  | (0.027) |  |
| France |  |  |  | -0.149^*^ |
|  |  |  |  | (0.078) |
| Germany |  |  |  | 0.160^**^ |
|  |  |  |  | (0.080) |
| UK |  |  |  | -0.023 |
|  |  |  |  | (0.073) |
| USA |  |  |  | 0.103 |
|  |  |  |  | (0.078) |
| Pol. attention: Class conflict | 0.028 |  |  |  |
|  | (0.018) |  |  |  |
| Pol. attention: Left neolib | -0.001 |  |  |  |
|  | (0.019) |  |  |  |
| Pol. attention: Strong + | -0.041 |  |  |  |
|  | (0.027) |  |  |  |
| Pol. attention: Don’t know | 0.041 |  |  |  |
|  | (0.033) |  |  |  |
| Pol. attention: Non-commit | -0.029 |  |  |  |
|  | (0.023) |  |  |  |
| Pol. attention: Acquiescer | 0.042 |  |  |  |
|  | (0.026) |  |  |  |
| Income: Class conflict |  | 0.003 |  |  |
|  |  | (0.034) |  |  |
| Income: Left neolib |  | -0.007 |  |  |
|  |  | (0.033) |  |  |
| Income: Strong + |  | -0.016 |  |  |
|  |  | (0.043) |  |  |
| Income: Don’t know |  | -0.039 |  |  |
|  |  | (0.067) |  |  |
| Income: Non-commit |  | -0.046 |  |  |
|  |  | (0.041) |  |  |
| Income: Acquiescer |  | 0.028 |  |  |
|  |  | (0.045) |  |  |
| Education: Class conflict |  |  | 0.012 |  |
|  |  |  | (0.045) |  |
| Education: Left neolib |  |  | 0.008 |  |
|  |  |  | (0.043) |  |
| Education: Strong + |  |  | 0.065 |  |
|  |  |  | (0.057) |  |
| Education: Don’t know |  |  | -0.085 |  |
|  |  |  | (0.080) |  |
| Education: Non-commit |  |  | 0.021 |  |
|  |  |  | (0.051) |  |
| Education: Acquiescer |  |  | -0.026 |  |
|  |  |  | (0.056) |  |
| France: Class conflict |  |  |  | -0.134 |
|  |  |  |  | (0.198) |
| Germany: Class conflict |  |  |  | -0.402^**^ |
|  |  |  |  | (0.203) |
| UK: Class conflict |  |  |  | -0.194 |
|  |  |  |  | (0.210) |
| USA: Class conflict |  |  |  | -0.126 |
|  |  |  |  | (0.211) |
| France: Left neolib |  |  |  | 0.202 |
|  |  |  |  | (0.140) |
| Germany: Left neolib |  |  |  | -0.110 |
|  |  |  |  | (0.136) |
| UK: Left neolib |  |  |  | 0.036 |
|  |  |  |  | (0.126) |
| USA: Left neolib |  |  |  | -0.002 |
|  |  |  |  | (0.129) |
| France: Strong + |  |  |  | 0.592^***^ |
|  |  |  |  | (0.228) |
| Germany: Strong + |  |  |  | 0.281^*^ |
|  |  |  |  | (0.168) |
| UK: Strong + |  |  |  | 0.257 |
|  |  |  |  | (0.166) |
| USA: Strong + |  |  |  | 0.054 |
|  |  |  |  | (0.148) |
| France: Don’t know |  |  |  | -0.070 |
|  |  |  |  | (0.283) |
| Germany: Don’t know |  |  |  | 0.173 |
|  |  |  |  | (0.310) |
| UK: Don’t know |  |  |  | 0.245 |
|  |  |  |  | (0.304) |
| USA: Don’t know |  |  |  | 0.038 |
|  |  |  |  | (0.300) |
| France: Non-commit |  |  |  | 0.389^**^ |
|  |  |  |  | (0.174) |
| Germany: Non-commit |  |  |  | -0.068 |
|  |  |  |  | (0.195) |
| UK: Non-commit |  |  |  | 0.102 |
|  |  |  |  | (0.173) |
| USA: Non-commit |  |  |  | 0.130 |
|  |  |  |  | (0.171) |
| France: Acquiescer |  |  |  | 0.119 |
|  |  |  |  | (0.253) |
| Germany: Acquiescer |  |  |  | -0.017 |
|  |  |  |  | (0.226) |
| UK: Acquiescer |  |  |  | 0.048 |
|  |  |  |  | (0.217) |
| USA: Acquiescer |  |  |  | -0.042 |
|  |  |  |  | (0.207) |
| N | 4393 | 4393 | 4393 | 4393 |
| R-squared | 0.158 | 0.157 | 0.156 | 0.162 |
| Adj. R-squared | 0.150 | 0.148 | 0.148 | 0.151 |
|  | | | | |
| ^***^p < .01; ^**^p < .05; ^*^p < .1  All models include controls for age, gender, equality values, social and political trust, labour force status, employment type, and the other variables listed in the table (income and education, as factors, political attention, country) that are not part of the interaction investigated in each given model. These variables are omitted and coefficients are suppressed in the table. | | | | |

# Alternative Analysis: No-Covariate Latent Class Analysis Models

As noted above, an alternative way to analyse the data is to construct the latent classes independently from modelling the links between covariates and class membership. Not only can we then use those classes to create analogous descriptions of the classes, but we can also use these classes as the central independent variables in the regression models for tax progressivity preferences.

Output from regressions using this approach can be seen below in Table A11. For reasons of space and simplicity, the table reports only those variables that appear in the latent class analyses (whether as classes or covariates). Model 1 shows the results from the simple model including only the latent classes, while model 2 is the model with a full set of controls (except party family), analogous to model 6 in Table A9. For ready comparison, the analogous coefficients from that model are reproduced in the final column of the table.

Overall, the substantive results are very similar under this alternative approach. The strong positive sum group remains significantly less supportive of progressive taxation, while the class conflict group, in contrast, endorses much greater progressivity, when compared to the (omitted) weak positive sum group. Equally, the don't know group endorse progressivity yet more strongly. Under the nine-class analysis the estimates for the left-neoliberal group become much more dependent on the inclusion or exclusion of covariates, as the instability of the coefficient across models 1 and 2 exemplifies. Such "sign switches" occur across analogous specifications to those in Table A9 as different sets of covariates are included (not shown).

The inferences we would draw from the sequential versus the simultaneous inclusion of covariates are thus the same: economic thinking, in the form of class conflict, positive sum, or disengagement, matters to progressivity preferences beyond its association with material interests or moral values. The minor fluctuations in the absolute magnitude of the estimated relationships encapsulate the slight variations in partitioning the relevant variance between the classes and covariates, because the estimated correlation between these differs somewhat under the two approaches.

| Table A11: Progressive taxation and latent classes from nine-class, no covariate model.  Baseline (omitted) categories for categorical covariates are: weak positive sum (economic thinking), Denmark (country), lowest quintile (income), incomplete secondary (education) and don't know (party vote). |
| --- |

|  | | **Tax progressivity preference** | | | | | |
| --- | --- | --- | --- | --- | --- | --- | --- |
|  | **Model 1** | | | **Model 2** | | | **Model 6, Table A9** |
|  | | |  |  | | (comparable to  Model 2) | |
| Strong + | -0.232^***^ | | | -0.218^***^ | | | -0.297^***^ |
|  | (0.054) | | | (0.053) | | | (0.057) |
| Left-neoliberal | -0.051 | | | -0.095^**^ | | | 0.015 |
|  | (0.048) | | | (0.048) | | | (0.045) |
| Class conflict | 0.368^***^ | | | 0.219^***^ | | | 0.212^***^ |
|  | (0.044) | | | (0.046) | | | (0.049) |
| Don't know | 0.174^*^ | | | 0.321^*^ | | | 0.356^***^ |
|  | (0.095) | | | (0.168) | | | (0.087) |
| Non-committal | -0.255^***^ | | | -0.223^***^ | | | -0.171^***^ |
|  | (0.057) | | | (0.061) | | | (0.055) |
| Acquiescer | 0.335^***^ | | | 0.204^***^ | | | 0.345^***^ |
|  | (0.049) | | | (0.050) | | | (0.063) |
| Rejection & conflict | 0.494^***^ | | | 0.154^*^ | | |  |
|  | (0.077) | | | (0.081) | | |  |
| No purpose | 0.244^***^ | | | 0.191^***^ | | |  |
|  | (0.055) | | | (0.065) | | |  |
| Income 2 |  | | | 0.021 | | | 0.030 |
|  |  | | | (0.037) | | | (0.037) |
| Income 3 |  | | | -0.002 | | | 0.004 |
|  |  | | | (0.037) | | | (0.037) |
| Income 4 |  | | | -0.077^**^ | | | -0.067^*^ |
|  |  | | | (0.039) | | | (0.039) |
| Income 5 |  | | | -0.095^**^ | | | -0.075^*^ |
|  |  | | | (0.042) | | | (0.043) |
| Secondary vocational |  | | | 0.139^***^ | | | 0.144^***^ |
|  |  | | | (0.044) | | | (0.044) |
| Secondary academic |  | | | 0.149^***^ | | | 0.156^***^ |
|  |  | | | (0.045) | | | (0.045) |
| Degree |  | | | 0.184^***^ | | | 0.194^***^ |
|  |  | | | (0.043) | | | (0.043) |
| France |  | | | 0.033 | | | 0.010 |
|  |  | | | (0.040) | | | (0.041) |
| Germany |  | | | 0.117^***^ | | | 0.089^**^ |
|  |  | | | (0.038) | | | (0.039) |
| UK |  | | | 0.041 | | | 0.032 |
|  |  | | | (0.038) | | | (0.038) |
| USA |  | | | 0.143^***^ | | | 0.132^***^ |
|  |  | | | (0.039) | | | (0.039) |
| Gender |  | | | -0.025 | | | -0.015 |
|  |  | | | (0.023) | | | (0.023) |
| Age |  | | | 0.007^***^ | | | 0.008^***^ |
|  |  | | | (0.001) | | | (0.001) |
| N | 5027 | | | 4393 | | | 4943 |
| R-squared | 0.052 | | | 0.159 | | | 0.156 |
| Adj. R-squared | 0.050 | | | 0.152 | | | 0.149 |
|  | | | |  | | | |
| ^***^p < .01; ^**^p < .05; ^*^p < .1 | | | | |  | | |

Model 2 and model 6 (A9) also include controls for political attention, social and political trust, values of equality, labour force status and employment type.

# Alternative Analysis: Zero-Sum Survey Item Regression Models

The payoff to the latent class analysis is its enabling us to compress multidimensional patterns in the data to the profiles of individual response patterns that best differentiate the groups that actually exist in the population. This multidimensionality comes in part from the desire to include don't know answers, as well as other types of non-response, as substance rather than nuisance in the responses. But it also results from the way that the zero- and positive-sum items (fail to) relate to one another.

That is, a priori, we might have thought that combining responses to the items in the battery could yield a general scale of zero-sum thinking versus positive-sum thinking. This would cleave more closely to the theoretical setup (although it raises problems of how to deal with non-response). However, as the main analysis indirectly indicates, trying to create such a scale would be trying to force a structure on the data that is not really there.

This can be seen in the low levels of reliability that would result from converting the different economic thinking variables into combined scales, as shown in Table A12.

Table A12: Reliability estimates (Cronbach's alpha) and confidence intervals for scales combining elements of the zero- and positive-sum item battery. Shaded cells indicate opposite-signed items, where one scale has been reversed.

|  |  | Positive sum conflict | Positive sum purpose | Positive sum labour markets | All items combined |
| --- | --- | --- | --- | --- | --- |
|  |  | 0.57  (0.55, 0.59) | | |  |
| Zero sum conflict | 0.5  (0.48, 0.53) | 0.2  (0.15, 0.25) |  |  |  |
| Zero sum purpose |  |  | 0.46  (0.43, 0.49) |  |  |
| Zero sum labour markets |  |  |  | 0.16  (0.12, 0.21) |  |
| All items combined |  |  | | | 0.36  (0.33, 0.39) |

Table A12 indicates that the scope for combining the various items in the zero- and positive-sum battery of items into a reliable scale for measuring a general zero to positive sum orientation is extremely limited. Combining the full set of six measures yields a scale with a reliability of only 0.36, well below a reasonable threshold for a reliable measure (usually taken to be 0.7). The positive sum items taken together are the most coherent, but even they have a reliability of below 0.6. The conflict between rich and poor, and the interpretation of labour market competition, though plausibly conceived of as the opposite ends of the conceptual scale running from a zero-sum to a positive-sum dominated view yield negligible levels of (negatively related) consistency, while the two items about what economics and economic policy are about yield a scale more readily interpretable as a propensity to express an opinion on what economics is for than a trade-off between the two goals, as agreement on one of these items is positively related to the other.

Is this a problem of the measurement instrument, or with the underlying conceptual expectation that these items should act together to describe a common underlying structure of economic thinking? There are a couple of reasons to think that it is likely the latter. First, considering zero sum thinking and positive sum thinking as two ends of a consistent scale in some ways presupposes the existence of a trade-off in reality -- for example, between equality and efficiency. While this relationship anticipated in (some) academic economic treatments (Okun 1975) it remains empirically contested even within economics (Berg 2018). Moreover, even if the existence of the trade-off were binding, in fact, if voters do not perceive it, there would be no need to expect it to be reflected in their thinking -- abandoning a pro-growth mindset *because* of its incompatibility with desired distributive goals would require a high level of awareness of this trade-off which we would not want to presuppose.

Empirically, then, we can see that for the subset of respondents that we might most expect it to, the combined battery does represent a scale with reasonable reliability: among those in the top income quintile, the top education group, and the top quintile of political attention -- the Zallerian elite, if you will -- the standardised Cronbach's alpha level is 0.67, almost at the threshold level (usually taken to be 0.7) for reliability. This also helps us to understand why scholars working on economic thinking may be looking for this kind of structure in popular economic thought, as scholars are demographically most similar to this group. Within this group, too, we see much stronger pairwise correlations between the zero-sum items and the positive-sum items (although even here there are only few and extremely weak negative correlations between the zero-sum and positive-sum items.

In the full sample, even the pairwise correlations between items are quite low. They are displayed in Figure A10 below. The strongest associations are between a zero-sum view of the conflict between rich and poor and of workers gaining employment in the labour market, on one hand; and between a positive sum view of new employment and of the overall purpose of economic policy being to increase the size of the pie (both of these pairs have a correlation of 0.4). Again, the positive correlation between the two "purpose" statements (r = 0.3) indicates that in contrast to the stylised discrepancy between expert and lay economic thinking, in the population as a whole it is consistent to claim both that economics and economic policy should aim at increasing the size of the pie, and that they should aim at managing the distribution of existing resources. A focus on distribution does not preclude attention to growth.

Figure A10: Correlation matrix for the six zero- and positive-sum survey items.

This context not only recommends changing the focus of the analysis, away from the stylised theoretical expectations about variables and towards the respondent-profile oriented approach of the latent class models (see 2a, above), and highlights again the tension between the theoretical expectations of zero-sum thinking, and the findings that this is not an obvious way in which these respondents seem to approach economics (see 1b above, but also 3a, below). It also makes the "traditional analyses" advocated by R1 harder to pursue, as there is not an obvious "zero-versus-positive sum-ness" element to popular thinking that can be readily operationalised.

However, it certainly is possible to consider the specific battery items individually in terms of their associations with tax progressivity preferences. The tables below present the results of these analyses in bivariate models (Table A13) and in models including the full suite of covariates used elsewhere in the paper (Table A14).

Table A13: Regression output for zero sum/positive sum battery items as individual determinants of progressivity preferences. Bivariate/no additional covariate models.

|  | | | | | | | |
| --- | --- | --- | --- | --- | --- | --- | --- |
|  | **Tax progressivity preference** | | | | | | |
|  | **Model 1** | **Model 2** | **Model 3** | **Model 4** | **Model 5** | **Model 6** | **Model 7** |
|  | | | | | | | |
| Zero sum conflict | 0.15^***^ |  |  |  |  |  | 0.16^***^ |
|  | (0.01) |  |  |  |  |  | (0.01) |
| Zero sum purpose |  | 0.01 |  |  |  |  | 0.01 |
|  |  | (0.01) |  |  |  |  | (0.01) |
| Zero sum labour markets |  |  | 0.02 |  |  |  | -0.06^***^ |
|  |  |  | (0.01) |  |  |  | (0.01) |
| Positive sum conflict |  |  |  | -0.10^***^ |  |  | -0.08^***^ |
|  |  |  |  | (0.01) |  |  | (0.01) |
| Positive sum purpose |  |  |  |  | -0.02^*^ |  | 0.0003 |
|  |  |  |  |  | (0.01) |  | (0.01) |
| Positive sum labour markets |  |  |  |  |  | -0.04^***^ | 0.03^*^ |
|  |  |  |  |  |  | (0.01) | (0.02) |
| N | 4891 | 4724 | 4780 | 4774 | 4671 | 4855 | 4319 |
| R-squared | 0.05 | 0.0003 | 0.0004 | 0.02 | 0.001 | 0.002 | 0.07 |
| Adj. R-squared | 0.05 | 0.0001 | 0.0002 | 0.02 | 0.0005 | 0.001 | 0.07 |
| ^***^p < .01; ^**^p < .05; ^*^p < .1 | | | | | | | |

These models indicate that the ideas that the fortunes of the rich come at the expense of others are the most clearly associated with demands for more progressive taxation, while agreement with the idea that what benefits the rich increases total wealth reduces support for progressivity. Ideas about the place of "the rich" and progressivity are not so surprising, and operate as expected. The rest of the positive sum items -- emphasising growth as the purpose of economic action and recognising positive-sum effects of employment -- are also negatively signed in the bivariate analyses, but these relationships are not robust to the simultaneous inclusion of all the battery items together (Model 7).

Table A14: Regression output for zero sum/positive sum battery items as individual determinants of progressivity preferences. All models with full set of covariates.

|  | | | | | | | |
| --- | --- | --- | --- | --- | --- | --- | --- |
|  | **Tax progressivity preferences** | | | | | | |
|  | **Model 1** | **Model 2** | **Model 3** | **Model 4** | **Model 5** | **Model 6** | **Model 7** |
|  | | | | | | | |
| Zero sum conflict | 0.08^***^ |  |  |  |  |  | 0.10^***^ |
|  | (0.01) |  |  |  |  |  | (0.01) |
| Zero sum purpose |  | 0.02^*^ |  |  |  |  | 0.01 |
|  |  | (0.01) |  |  |  |  | (0.01) |
| Zero sum labour markets |  |  | 0.0004 |  |  |  | -0.04^***^ |
|  |  |  | (0.01) |  |  |  | (0.01) |
| Positive sum conflict |  |  |  | -0.04^***^ |  |  | -0.03^***^ |
|  |  |  |  | (0.01) |  |  | (0.01) |
| Positive sum purpose |  |  |  |  | 0.01 |  | 0.005 |
|  |  |  |  |  | (0.01) |  | (0.01) |
| Positive sum labour markets |  |  |  |  |  | 0.01 | 0.03^**^ |
|  |  |  |  |  |  | (0.01) | (0.02) |
| N | 4327 | 4224 | 4251 | 4253 | 4181 | 4314 | 3914 |
| R-squared | 0.18 | 0.17 | 0.17 | 0.17 | 0.17 | 0.17 | 0.19 |
| Adj. R-squared | 0.18 | 0.16 | 0.16 | 0.16 | 0.16 | 0.16 | 0.18 |
|  | | | | | | | |
| ^***^p < .01; ^**^p < .05; ^*^p < .1  Additional controls in all models: gender, age, labour force status, employment type, social and political trust, justice-equality beliefs, income, education, party vote, country. | | | | | | | |

1. Because the coefficient estimates on covariates come from multinomial logistic models, other variables' levels must be specified and held constant to generate the predicted probabilities. These are set to: female, 48 years old, country equal to UK, income equals median (group 3) (panel (b)) and education the modal level (secondary academic) (panel (a)). [↑](#footnote-ref-1)
